# Study of Microbial Contamination of Mobile Phones of Dental and Medical Students and Its Potential Epidemiological Implications for Microbial Transmission

**DOI:** 10.3390/life16091394

**Published:** 2026-08-24

**Authors:** Veselina Kondeva, Velina Stoeva, Yordan Kalchev, Rumyana Stoyanova

**Affiliations:** 1 Department of Pediatric Dentistry, Faculty of Dental Medicine, Medical University of Plovdiv, 4002 Plovdiv, Bulgaria; veselina.kondeva@mu-plovdiv.bg; 2 Department of Epidemiology and Disaster Medicine, Faculty of Public Health, Medical University of Plovdiv, 4002 Plovdiv, Bulgaria; 3 Department of Medical Microbiology and Immunology, Faculty of Medicine, Medical University of Plovdiv, 4002 Plovdiv, Bulgaria; yordan.kalchev@mu-plovdiv.bg; 4 Division of Innovative Diagnostic Methods, Research Institute at Medical University of Plovdiv, Medical University of Plovdiv, 4002 Plovdiv, Bulgaria; 5 Department of Health Management and Health Economics, Faculty of Public Health, Medical University of Plovdiv, 4002 Plovdiv, Bulgaria

**Keywords:** mobile phones, microbial contamination, healthcare students, hand hygiene, infection control

## Abstract

Background: Mobile phones are widely used by healthcare students during clinical training and may act as potential reservoirs for pathogenic microorganisms. Objectives: This study aimed to investigate and evaluate the role of mobile phones as epidemiological factors in the transmission of microorganisms in medical and dental practice. Methods: A cross-sectional epidemiological and microbiological study was conducted between September 2025 and April 2026. A total of 125 mobile phones belonging to fourth-year dental students and fifth-year medical students were examined. Samples were collected from the entire phone surface and cultured on blood agar, eosin–methylene blue agar, and Candida chromogenic agar. Participants also completed a structured questionnaire assessing demographic characteristics, clinical experience, hand hygiene practices, and mobile phone use and cleaning habits. Results: No microbial growth was detected in 52.8% of samples on blood agar, while 44.8% showed growth below 10^5^ CFU of coagulase-negative staphylococci and 2.4% showed growth ≥10^5^ CFU. Minimal growth was detected on eosin–methylene blue agar (0.8%). Fungal contamination was identified in 2.4% of samples, exclusively among students who reported touching their phones with contaminated gloves. Statistically significant associations were observed between microbiological growth and students’ specialty, duration of clinical experience, and contact with mobile phones while wearing contaminated gloves (*p* < 0.05). Conclusions: Touching mobile phones with contaminated gloves was significantly associated with microbial growth. These findings emphasize the need for targeted infection control education and stricter guidelines regarding mobile device use during clinical training.

## 1. Introduction

Mobile phones have been an integral part of the daily professional activities of medical and dental professionals for decades. Their advantages are many, and nowadays, personal and professional communication between members of healthcare teams is unthinkable without them. They also provide quick access to medical information and literature. Modern medical and dental practice increasingly relies on mobile devices, but without regular and appropriate disinfection, these devices serve as potential epidemiological factors for transmitting microorganisms among medical and dental teams. Mobile phones are a potential reservoir and vehicle for the transmission of microorganisms, both in healthcare facilities and in the community. Their frequent handling, infrequent cleaning, and use during patient care create conditions for the accumulation and spread of pathogenic bacteria [1,2,3].

The role of mobile phones as potential epidemiological factors in the transmission of infections, both between members of the medical and dental staff and between staff and patients, has been examined in several studies. Mobile phones are devices that are classified as high-touch surfaces. They are frequently handled by medical and dental professionals, potentially also patients, and surfaces in medical and dental environments. Various studies have found a variety of pathogens and potentially pathogenic microorganisms on the surface of mobile devices, including *Staphylococcus aureus*, methicillin-resistant *Staphylococcus aureus* (MRSA), coagulase-negative staphylococci, *Escherichia coli*, *Pseudomonas aeruginosa*, *Klebsiella pneumoniae complex*, as well as various viruses and fungi [4,5,6].

The specifics of work in medical and dental practice are such that there is a constant risk of contact with biological fluids (blood, saliva, etc.) from patients and contamination with them of various surfaces, instruments, equipment, including mobile phones. In most dental procedures, as well as in some medical procedures, aerosols are generated through the use of high-speed instruments, facilitating the dissemination of microorganisms to distant areas of the clinical environment and contributing to the contamination of surfaces beyond the immediate vicinity of the patient. These aerosols may carry microorganisms originating, for example, from the patient’s oral cavity and facilitate their deposition on various surfaces, including mobile phones. Insufficient or suboptimal hand hygiene and the lack of regular disinfection of mobile devices may further contribute to cross-contamination and the spread of infection [1,7,8,9,10].

Worryingly, several studies have reported the presence of multidrug-resistant microorganisms on mobile phones. A recent 30-month cross-sectional study conducted at a university hospital in Germany analyzed 232 mobile phones belonging to healthcare workers (HCWs) and 241 belonging to non-HCWs. The study found that the prevalence of resistant microorganisms, including methicillin-resistant *Staphylococcus aureus* (MRSA) and vancomycin-resistant enterococci, was significantly higher on HCW phones than on non-HCW phones (15.1% vs. 0.4%, *p* < 0.001) [11].

Mobile devices may therefore serve as reservoirs for highly antibiotic-resistant microorganisms and may contribute to the cross-transmission of resistant strains among healthcare workers, patients, and contaminated surfaces [12,13,14].

The World Health Organization recognizes antimicrobial resistance as one of the major threats to public health. In this context, mobile phones may represent a potentially insufficiently controlled link in the epidemiological chain of transmission of antimicrobial-resistant microorganisms. Regular and thorough disinfection of mobile devices, combined with strict adherence to hand hygiene practices, is considered an important measure for limiting the spread of resistant bacteria in healthcare settings [1,15,16,17].

**Aim:** to investigate and evaluate the role of mobile phones as epidemiological factors in the transmission of microorganisms in medical and dental practice.

## 2. Materials and Methods

A cross-sectional epidemiological and microbiological study was conducted between September 2025 and April 2026 to investigate microorganisms potentially transferred to the mobile phone surfaces of medical and dental students through contact with the skin, oral cavity, environment, or other frequently touched surfaces. The study included fourth-year dental students and fifth-year medical students at the Medical University-Plovdiv, Plovdiv, Bulgaria.

A total of 125 personal mobile phones were included in the study.

Before sample collection, all participants were informed about the aims and objectives of the study, the expected benefits, and their right to withdraw at any time without providing a reason. Written informed consent was obtained from all participants.

To ensure anonymity and traceability of the results, each participant was assigned an individual alphanumeric identification code. The same code was used to link the microbiological sample with the corresponding questionnaire data and to allow participants to access their individual laboratory results after completion of the analysis. Questionnaire data and microbiological results were analyzed in aggregated form and used solely for scientific purposes.

### 2.1. Questionnaire

All participants who provided written informed consent completed an anonymous, structured questionnaire developed by the research team specifically for this study. The questionnaire was designed to collect participants’ demographic characteristics and clinical experience and to assess their knowledge and routine practices related to hand hygiene and mobile phone use and disinfection during clinical activities, as well as their awareness of the potential role of mobile phones in the transmission of microorganisms.

The questionnaire included items addressing age, sex, field of study, year of study, duration of clinical experience, frequency and knowledge of hand hygiene practices, use of gloves, handling of mobile phones during patient care, frequency and methods of mobile phone cleaning, and awareness of mobile phones as potential reservoirs and vehicles for the transmission of microorganisms.

The questions addressing hand hygiene practices, mobile phone use and disinfection, and awareness of microbial transmission are presented in Table 2. Questionnaire data were used solely for scientific purposes and analyzed in aggregated form.

### 2.2. Microbiological Sampling and Analysis

Microbiological samples were collected from the entire external surface of each mobile phone using dry, sterile cotton swabs applied with firm pressure. The swabs were subsequently placed in a gel-based transport medium (Amies; Biolife Italiana S.r.l., Milan, Italy). Sample collection was performed without direct contact between the collector’s hands and the mobile phones in order to minimize the risk of external contamination. All specimens were processed within 24 h of collection in the microbiology laboratory of the Medical University–Plovdiv.

Laboratory processing was performed using a biosafety cabinet and in accordance with standard aseptic procedures to minimize the possibility of contamination during microbiological analysis. The samples were inoculated onto 5% sheep blood agar, eosin-methylene blue (EMB) agar, and Candida chromogenic agar. The culture media were incubated at 36–37 °C for up to 48 h, while the chromogenic fungal medium was incubated for up to 5 days.

The microbiological analysis was designed to recover non-fastidious aerobic and facultatively anaerobic microorganisms potentially originating from the skin, oral cavity, environment, or other frequently contacted surfaces. This included microorganisms commonly associated with resident skin microbiota, as well as microorganisms that may be introduced through transient contamination during handling, such as coagulase-negative staphylococci (CoNS). No separate assessment of the participants’ skin microbiota was performed.

Particular attention was given to microorganisms of potential clinical relevance, including *Staphylococcus aureus*, *Enterococcus* spp., and Gram-negative enteric microorganisms. These microorganisms were considered relevant because mobile phones used in clinical settings may become contaminated through contact with healthcare workers’ hands, patients, and contaminated environmental surfaces and may subsequently act as potential reservoirs or vehicles for microbial transmission. *Candida* spp. were also investigated because these yeasts can colonize the skin and mucosal surfaces and may persist on frequently handled surfaces.

Bacterial identification was performed using routine manual biochemical tests. *Candida* spp. were identified based on the characteristic colour of colonies on the chromogenic medium.

### 2.3. Statistical Analysis

Data was analyzed using descriptive and analytical statistical methods. Quantitative variables were expressed as mean values with corresponding standard deviations, while qualitative variables were presented as absolute numbers and percentages, allowing for a comprehensive characterization of the study sample.

The distribution and type of variables were taken into consideration when selecting the appropriate statistical tests. Associations between categorical variables were evaluated using the Pearson chi-square test, while Fisher’s exact test was applied when the expected frequencies were insufficient for the reliable application of the chi-square test. Spearman’s rank correlation coefficient was used to assess the strength and direction of the association between ordinal variables, where appropriate.

A *p*-value < 0.05 was considered statistically significant. Statistical analyses were performed using SPSS version 23 (IBM Corp., Armonk, NY, USA) and Microsoft Excel 2016.

## 3. Results

The distribution of students by gender shows a predominance of women—64.0% (*n* = 80), while men are 36.0% (*n* = 45). The average age of the people participating in the study, from whose mobile phones samples were taken for microbiological examination, is 22.74 ± 0.753 years. By specialty, the participants are distributed as follows: 24.0% (*n* = 30) are medical students, and 76.0% (*n* = 95) are dental students.

In terms of clinical experience, 74.4% (*n* = 93) of the students have 2 years of clinical practice, and 25.6% (*n* = 32) have 3 years. This distribution reflects differences in curricula, with medical students mainly in their fifth year and, accordingly, with longer clinical experience, while dental students are mainly in their fourth year. This difference in the stages of the two students’ years is due to an interruption in their studies and subsequent re-enrollment in the fourth year.

### 3.1. Microbiological Findings in Mobile Phone Testing

The distribution of results according to isolates on Blood agar, Eosin-methylene blue agar, and Candida chromogenic agar is presented in Table 1.

The results show that in more than half of the examined mobile phones (*n* = 66; 52.8%), no growth of microorganisms was seen on 5% sheep blood agar (Table 1). When cultivated on eosin-methylene blue agar, the majority of samples (*n* = 124; 99.2%) showed no microbial growth of Gram-negative enteric bacteria, with only one sample showing minimal growth below 10^5^ CFU (*n* = 1; 0.8%). The analysis of the samples cultured on Candida chromogenic agar showed no fungal growth in 97.6% of cases (*n* = 122), while Mold growth was detected in 2.4% (*n* = 3) (Table 1).

### 3.2. Hygiene Habits and Use of Mobile Phones During Clinical Activities

Table 2 presents the distribution of students’ responses related to hygiene practices during clinical work with patients.

**Table 2:** Distribution of students’ responses regarding hygienic hand disinfection skills during clinical practice.

Questions		*n*	%
How often do you perform hygienic hand disinfection while working with patients?	Follow up on each patient’s work	39	31.2
	Before working with each patient; Only if hands are visibly dirty	9	7.2
	Before and after working with each patient	69	55.2
	Total	125	100.0
What is the recommended minimum time interval for performing hygienic disinfection?	5 s	19	15.2
	10 s	46	36.8
	20–30 s	60	48.0
	Total	125	100.0
Does wearing gloves replace the need for hand disinfection?	yes	0	0.0
	no	125	100.0
	Total	125	100.0
Do you touch your personal mobile phone with contaminated gloves while working with patients?	yes	36	28.8
	no	89	71.2
	Total	125	100.0
If your answer is “yes”, for what reason do you most often have to use your mobile phone at work?	to answer emergency calls from patients	13	10.4
	to record patients’ appointments	1	0.8
	to look at radiographs	12	9.6
	to answer emergency calls and register patients	1	0.8
	to answer emergency calls from patients and to look at radiographs	5	4.0
	to answer emergency calls, register patients, and review radiographs	5	4.0
	Missing	88	70.4
	Total	125	100.0
How often do you clean your phone?	Every day	7	5.6
	Once a week	32	25.6
	Rarely	77	61.6
	Never	9	7.2
	Total	125	100.0
What do you use to clean it?	Alcohol disinfectant	43	34.4
	Dry cloth	12	9.6
	Wet wipes	57	45.6
	I do not clean it	6	4.8
	Alcohol disinfectant and wet wipes	6	4.8
	Alcohol disinfectant, dry and wet wipes	1	0.8
	Total	125	100.0
Do you think your phone could be a reservoir of pathogenic microorganisms?	yes	124	99.2
	no	1	0.8
	Total	125	100.0
According to the report, it is possible to transmit microorganisms such as *Staphylococcus aureus*, *Escherichia coli*, and *Pseudomonas aeruginosa* through a mobile phone.	yes	124	99.2
	no	1	0.8
	Total	125	100.0

The survey results showed differences in students’ reported hand hygiene practices during clinical work with patients (Table 2). The largest proportion of participants, 55.2% (*n* = 69), reported that they disinfect their hands both before and after contact with the patient, which corresponds to the recommended good hygiene practices. However, 31.2% (*n* = 39) disinfect their hands only after working with the patient, 7.2% (*n* = 9)—only before contact with the patient, and 6.4% (*n* = 8) indicated that they disinfect only when hands are visibly dirty. Knowledge regarding the recommended use of alcohol-based disinfectants also shows significant variability. Only 48.0% (*n* = 60) of the students indicated the correct minimum time interval of 20–30 s, while the rest underestimated the required time, with 36.8% (*n* = 46) considering 10 s to be sufficient and 15.2% (*n* = 19) considering only 5 s. These results question the effectiveness of actual disinfection, even when it is applied regularly.

All the students surveyed (100%; *n* = 125) agreed that wearing gloves does not replace hygienic hand disinfection. However, 28.8% (*n* = 36) admitted to touching their personal mobile phone with contaminated gloves during clinical work, which poses a potential risk of cross-contamination. Among the main reasons for using a mobile phone in this context are answering emergency calls from patients and displaying radiographic images, indicated by the majority of respondents (see Table 2).

Regarding the maintenance of the hygiene of mobile devices, the results show an unfavourable trend. Only 5.6% (*n* = 7) of the students clean their phone every day, and 25.6% (*n* = 32)—once a week, while the majority, 61.6% (*n* = 77) reported that they clean it rarely, and 7.2% (*n* = 9)—never. Although 34.4% (*n* = 43) use alcohol disinfectant, a significant proportion relies on ineffective methods such as cleaning with a dry or wet cloth. Despite the identified gaps in hygiene practices, almost all participants, 99.2% (*n* = 124) are clear that the mobile phone can serve as a reservoir of pathogenic microorganisms and that it is possible to transfer clinically significant bacteria. Relationships between microbiological results and demographic and behavioural factors.

The analysis of the relationships between microbiological results and the study of demographic and behavioural factors shows the presence of statistically significant relationships for some of the variables, while for others, such a relationship is not established.

### 3.3. Relationship Between the Specialty of the Students and Microbiological Growth on Blood Agar

A statistically significant relationship was established between the characteristics of the students (medicine and dentistry) and the results of the blood agar cultures (χ^2^ = 7.993; *p* = 0.018).

Among dental students, the proportion of samples without microbiological growth is more than (58.9%), while among medical students, growth below 10^5^ CoNS is often observed (66.7%). Growth above 10^5^ CoNS is established only among dental students (3.2%). The data show different microbiological contamination related to the specialty, which suggests an influence on the specifics of clinical work and contacts during training (see Table 3).

### 3.4. Relationship Between Clinical Experience and Microbiological Growth on Blood Agar

The analysis revealed a statistically significant relationship between the duration of clinical experience and the results of the cultural study on blood agar (χ^2^ = 7.961; *p* = 0.019).

In students with two years of clinical experience, lack of growth was more often registered (59.1%), while in those with three years of experience, the proportion of samples with growth below 10^5^ CoNS was higher (65.6%) (see Table 3).

### 3.5. Relationship Between Touching a Mobile Phone with Contaminated Gloves and Microbiological Growth on Blood Agar

The most pronounced relationship in the study was found between touching a personal mobile phone with contaminated gloves during clinical work and microbiological growth on blood agar (χ^2^ = 26.558; *p* < 0.001).

In students who do not touch their phone with contaminated gloves, in 66.3% of cases no microbiological growth was recorded. In contrast, in individuals who touch their mobile phone during work, growth below 10^5^ CoNS was significantly more often detected (72.2%), as well as all cases of growth above 10^5^ CoNS. Spearman’s rank correlation also indicated a moderate negative association (r = −0.446, *p* < 0.001), which confirms the influence of this behaviour on microbiological contamination (see Table 3).

### 3.6. Relationship Between Touching a Mobile Phone with Contaminated Gloves and Fungal Growth on Candida Chromogenic Agar

A statistically significant relationship was also found between touching a mobile phone with contaminated gloves and fungal growth on Candida chromogenic agar (χ^2^ = 7.599; *p* = 0.006; Fisher’s exact test *p* = 0.022). All three cases of recorded fungal growth were found in students who touched their mobile phones during clinical work, while none of the cases of fungal growth were reported in individuals who did not perform this behaviour (see Table 4). These findings further support the potential role of mobile devices as reservoirs of microorganisms in case of non-compliance with hygiene rules.

### 3.7. Lack of Statistically Significant Relationships

No statistically significant relationships were found between microbiological results and the majority of hygiene habits studied, including frequency of hand disinfection and cleaning of mobile phones (*p* > 0.05). These results indicate that demographic characteristics and declared hygiene practices alone do not significantly influence microbiological contamination in the study sample.

## 4. Discussion

Mobile phones are frequently used in healthcare settings, carried between offices and clinical areas, and are rarely subjected to routine disinfection. This practice creates favorable conditions for the accumulation and transmission of microorganisms among healthcare professionals, patients, and the hospital environment. The issue is highly relevant and widely discussed in scientific literature, and the findings observed in the present study are consistent with those reported by other research groups. Numerous studies have investigated the role of mobile phones as potential vectors of nosocomial infections, demonstrating that devices used by healthcare workers are often colonized with potentially pathogenic microorganisms due to frequent handling and insufficient disinfection [18,19,20]. Although establishing a direct cause-and-effect relationship remains challenging, many authors emphasize the importance of incorporating mobile device cleaning into standard hand hygiene practices [21].

Several studies have reported high contamination rates of healthcare workers’ mobile phones, including the presence of multidrug-resistant pathogens. One study found bacterial contamination in 84% of mobile phones, with approximately 80% of the isolated microorganisms exhibiting multidrug resistance [22]. Similar findings have been reported in hospital settings across different regions, highlighting the persistent role of mobile devices as reservoirs of infection despite increased awareness of cross-contamination risks [23].

Systematic reviews and meta-analyses further confirm the widespread nature of this problem. A meta-analysis involving 26 studies and 2887 healthcare workers reported bacterial contamination in approximately 84.5% of examined mobile phones, with coagulase-negative staphylococci, *Staphylococcus aureus*, and *Escherichia coli* being the most frequently isolated microorganisms [17]. Another comprehensive review analyzing studies from multiple countries reported an overall contamination rate of approximately 68% [7].

The findings of the present study are in line with these reports. Coagulase-negative staphylococci (CoNS) were the most commonly isolated microorganisms, detected at contamination levels below 10^5^ in 44.8% of samples and at levels ≥10^5^ in 2.4%. Lactobacilli (<10^5^ CFU) were identified in 0.8% of samples, while growth on Candida chromogenic agar was observed in 2.4%. Although CoNS are considered part of the normal skin microflora, their presence on mobile phones is undesirable and may serve as an indicator of inadequate or insufficient disinfection. Under certain conditions, these microorganisms may act as opportunistic pathogens. Additionally, the growth detected on eosin-methylene blue agar may indicate the presence of Gram-negative microorganisms and warrants attention to hand hygiene and environmental contamination.

In dental practice, the risk of contamination may be even higher due to the continuous generation of aerosols containing microorganisms from the patient’s oral cavity. Studies involving dental professionals have demonstrated significantly higher levels of microbial contamination of mobile phones compared to non-medical control groups. This contamination has been associated with frequent device handling during clinical work and suboptimal hand hygiene practices [24]. Despite the recognized role of aerosols in disseminating microorganisms throughout the clinical environment, the results of the present analysis indicate greater contamination of personal mobile phones among medical students. Specifically, no microbial growth was detected in only 33.3% of medical students’ phones, compared to 58.9% of dental students’ devices. This difference may reflect differences in clinical activities, patterns of mobile phone use, or other unmeasured factors; however, these explanations could not be established in the present study.

Previous studies have shown that only a small proportion of healthcare workers disinfect their mobile phones regularly. In one study approximately 15% reported disinfecting their phones at least once per week, while only 8% performed hand hygiene immediately after phone use during work [25]. Similarly, the survey conducted among students in the present study revealed suboptimal mobile phone disinfection practices. Only 5.6% of respondents reported daily disinfection of their devices, and 25.6% disinfected them once per week, whereas the majority (61.6%) reported rare disinfection.

Overall, these findings demonstrate persistent neglect of mobile devices as potential epidemiological factors in the transmission of healthcare-associated infections. Whenever possible, mobile phone use during clinical work should be limited, and when unavoidable, strict disinfection protocols should be applied. Proper hand hygiene remains the most effective measure for preventing nosocomial infections and interrupting the chain of microorganism transmission within healthcare facilities. Despite increased theoretical awareness among medical students, previous research indicates that knowledge does not always translate into appropriate practical behavior. One of the most frequently overlooked components of infection control continues to be the hygiene of mobile phones [26,27].

### 4.1. Recommendations for Infection Prevention and Control and Methods for Disinfecting Mobile Phones

The most commonly recommended disinfectant for mobile phones is 70% ethyl or isopropyl alcohol, which has been shown to significantly reduce bacterial load following application. The use of alcohol-based wipes is practical and easy to implement in daily clinical practice [26].

Despite the high levels of microbial contamination reported in the literature, the cleaning and disinfection of mobile phones may not always be adequately addressed within infection prevention and control practices [17]. Therefore, personal mobile devices should be explicitly considered in infection prevention and control programs. Key recommendations include regular disinfection of mobile phones, strict hand hygiene before and after device use, limiting mobile phone use in high-risk clinical areas, and providing periodic training for healthcare personnel. The integration of these preventive measures into routine medical and dental practice may substantially reduce the risk of pathogen transmission and enhance the safety of both patients and healthcare professionals.

Adherence to mobile device disinfection protocols aims to minimize the risk of microbial contamination and to prevent cross-transmission of infections between healthcare personnel and patients. Compliance with these protocols should be ensured among all medical and dental professionals, including doctoral students, who use mobile phones in healthcare settings.

An essential component of protocol implementation is the consistent performance of hand hygiene procedures in conjunction with mobile phone use.

**Frequency of disinfection**
  - At the beginning and end of each work shift;
  - After patient contact;
  - After contamination with blood, saliva, or other biological materials.
**Additional recommendations**
  - Mobile phones should not be used during invasive procedures;
  - Contact with mobile phones while wearing contaminated gloves should be avoided;
  - Mobile phones should be formally incorporated into the infection prevention and control programs of healthcare facilities.

### 4.2. Strengths

The main strengths of this study are the combination of objective microbiological sampling with questionnaire data, the inclusion of medical and dental students who have clinical contact with patients, the use of three types of culture media to facilitate the recovery of different groups of microorganisms, and the application of appropriate statistical methods. The study also provides both microbiological findings and information on participants’ self-reported hand hygiene and mobile phone handling practices, allowing these aspects to be considered together.

### 4.3. Limitations

Several limitations should be considered when interpreting the findings. First, the cross-sectional design does not allow causal relationships to be established. The study identifies microbial contamination of mobile phones and reports associations with self-reported hygiene and handling practices, but it cannot directly demonstrate that touching a mobile phone with contaminated gloves caused the observed contamination.

Second, the study included 125 mobile phones belonging to medical and dental students from a single institution. Therefore, the findings may not be generalizable to all healthcare professionals, healthcare settings, or other institutions and geographical locations. Larger multicentre studies, including different categories of healthcare professionals would be valuable to confirm and extend these findings.

Third, information on the frequency of hand disinfection, mobile phone cleaning, and phone use with contaminated gloves was obtained through a self-reported questionnaire and may therefore be subject to recall and social desirability bias. Consequently, the reported practices may not fully reflect participants’ actual behavior during clinical activities.

Finally, microbiological identification was based on phenotypic and routine biochemical methods, and molecular or genetic confirmation of the identified microorganisms was not performed. Therefore, species-level identification of some isolates, particularly coagulase-negative staphylococci and other groups for which molecular confirmation could provide greater taxonomic resolution, may be limited.

## 5. Conclusions

The study demonstrated the presence of microorganisms on the mobile phones of medical and dental students, with the findings indicating frequent microbial contamination of these devices. The reported use of mobile phones with contaminated gloves and insufficient cleaning practices were identified as important factors associated with the potential for contamination. Differences according to specialty and duration of clinical experience suggest that clinical exposure and work-related practices may also contribute to contamination patterns.

These findings highlight the potential role of mobile phones as reservoirs and possible vehicles for microbial transmission in clinical environments. Although almost all students reported awareness of the risk of infection transmission through mobile devices, mobile phones were often not cleaned sufficiently. Regular disinfection of mobile devices, strict hand hygiene, and limiting mobile phone use during patient care are therefore recommended.

Adherence to appropriate mobile phone disinfection practices should be considered as an important component of infection prevention and control programs in healthcare facilities and as part of standard precautions. The combination of proper hand hygiene, limiting the use of mobile devices during clinical activities, and the use of appropriate disinfectants may help reduce the potential risk of microbial transmission. Increasing awareness of mobile phones as potential reservoirs of microorganisms may support the development of effective infection prevention policies in medical and dental practice.

## Figures and Tables

**Table 1:** Distribution of microorganisms according to the growth of microorganisms on different growth media.

Culture Medium	Microbiological Result					
5% Sheep Blood Agar	No Growth		Growth < 10^5^ CoNS		Growth ≥ 10^5^ CoNS	
	*n*	%	*n*	%	*n*	%
	66	52.8	56	44.8	3	2.4
Eosin-methylene blue agar	No Growth		Growth < 10^5^		Growth ≥ 10^5^	
	*n*	%	*n*	%	*n*	%
	124	99.2	1	0.8	0	0.0
Candida Chromogenic Agar	No Growth		Yeast Growth Present		Mold Growth Present	
	*n*	%	*n*	%	*n*	%
	122	97.6	0	0.0	3	2.4

**Table 3:** Associations between blood agar growth and selected demographic and behavioral factors.

Demographic and Behavioral Factors		Blood Agar			Total *n* (%)	*p*
		No Growth *n* (%)	Growth < 10^5^ CoNS *n* (%)	Growth ≥ 10^5^ CoNS *n* (%)		
Student	Medical student	10 (33.3)	20 (66.7)	0 (0.0)	30 (100.0)	0.018
	Dental student	56 (58.9)	36 (37.9)	3 (3.2)	95 (100.0)	
	Total	66 (52.8)	56 (44.8)	3 (2.4)	125 (100.0)	
How many years of clinical experience do you have?	2 years	55 (59.1)	35 (37.6)	3 (3.2)	93 (100.0)	0.019
	3 years	11 (34.4)	21 (65.6)	0 (0.0)	32 (100.0)	
	Total	66 (52.8)	56 (44.8)	3 (2.4)	125 (100.0)	
Do you touch your personal mobile phone with contaminated gloves while working with patient	yes	7 (19.4)	26 (72.2)	3 (8.3)	36 (100.0)	0.000
	no	59 (66.3)	30 (33.7)	0 (0.0)	89 (100.0)	
	Total	66 (52.8)	56 (44.8)	3 (2.4)	125 (100.0)	

**Table 4:** Statistically significant associations between Candida chromogenic agar results and behavioral factors.

Behavioral Factor		Candida Chromogenic Agar		Total *n* (%)	*p*
		No Growth *n* (%)	There Is Mold Growth *n* (%)		
Do you touch your personal mobile phone with contaminated gloves while working with patients?	yes	33 (91.7)	3 (8.3)	36 (100.0)	0.022
	no	89 (100.0)	0 (0.0)	89 (100.0)	
	Total	122 (97.6)	3 (2.4)	125 (100.0)	

## Data Availability

The original contributions presented in this study are included in the article. Further inquiries can be directed at the corresponding author.
